# A cross sectional analysis of behaviors related to operating gas stoves and pneumonia in U.S. children under the age of 5

**DOI:** 10.1186/s12889-015-1425-y

**Published:** 2015-02-04

**Authors:** Eric S Coker, Ellen Smit, Anna K Harding, John Molitor, Molly L Kile

**Affiliations:** College of Public Health and Human Sciences, Oregon State University, Milam Hall, Corvallis, OR 97331 USA

**Keywords:** Gas stove, Heating, Ventilation, Pneumonia, Respiratory infection, NHANES

## Abstract

**Background:**

Poorly ventilated combustion stoves and pollutants emitted from combustion stoves increase the risk of acute lower respiratory illnesses (ALRI) in children living in developing countries but few studies have examined these issues in developed countries. Our objective is to investigate behaviors related to gas stove use, namely using them for heat and without ventilation, on the odds of pneumonia and cough in U.S. children.

**Methods:**

The National Health and Nutrition Examination Survey (1988–1994) was used to identify children < 5 years who lived in homes with a gas stove and whose parents provided information on their behaviors when operating their gas stoves and data on pneumonia (N = 3,289) and cough (N = 3,127). Multivariate logistic regression models were used to examine the association between each respiratory outcome and using a gas stove for heat or without ventilation, as well as, the joint effect of both behaviors.

**Results:**

The adjusted odds of parental-reported pneumonia (adjusted odds ratio [aOR] = 2.08, 95% confidence interval [CI]: 1.08, 4.03) and cough (aOR = 1.66, 95% CI: 1.14, 2.43) were higher among children who lived in homes where gas stoves were used for heat compared to those who lived in homes where gas stoves were only used for cooking. The odds of pneumonia (aOR = 1.76, 95% CI: 1.04, 2.98), but not cough (aOR = 1.23, 95% CI: 0.87, 1.75), was higher among those children whose parents did not report using ventilation when operating gas stoves compared to those who did use ventilation. When considering the joint association of both stove operating conditions, only children whose parents reported using gas stoves for heat without ventilation had significantly higher odds of pneumonia (aOR = 3.06, 95% CI: 1.32, 7.09) and coughing (aOR = 2.07, 95% CI: 1.29, 3.30) after adjusting for other risk factors.

**Conclusions:**

Using gas stoves for heat without ventilation was associated with higher odds of pneumonia and cough among U.S. children less than five years old who live in homes with a gas stove. More research is needed to determine if emissions from gas stoves ventilation infrastructure, or modifiable behaviors contribute to respiratory infections in children.

## Background

In the United States, acute lower respiratory infections (ALRI), which include bronchitis, influenza and pneumonia, area leading cause of hospitalizations among children under five years of age. In 2010, it was estimated that there were 313,322 hospitalizations for new cases of ALRI in the United States among children in this age group [1]. The introduction of pneumococcal and Haemophilus influenza type b vaccines over the past decade significantly decreased overall pneumonia incidence [2,3], yet the rates for non-vaccine serotypes of pneumonia have largely remained unchanged among children under five years of age [2,4]. Early childhood ALRIs may also have persistent effects on respiratory health and have been linked with an increased risk of asthma later in childhood [5-7]. Consequently, it remains important to identify environmental factors that contribute to, or that can prevent, ALRI in young children.

Researchers have identified many risk factors for ALRI in young children including low birth weight, lack of breastfeeding, crowded housing conditions, exposure to tobacco smoke, low household income, low maternal educational attainment, day care attendance, and incomplete immunizations [1,8]. Indoor air pollution from combustion stoves and heaters are also known risk factors for ALRI in young children [1,8,9]. The vast majority of the data supporting the association between ALRI and indoor air pollution has been collected in developing countries and has been attributed to burning solid fuels (e.g. wood, coal, and charcoal) [9-11] indoors for cooking and heating. However, the health impact from indoor air pollution generated from gas stoves on respiratory infections in children living in developed countries is less understood [12].

Data from indoor air pollution studies conducted in developed countries indicate that using gas stoves leads to elevated concentrations of nitrogen dioxide and particulate matter inside homes [12-17]. Studies have also reported a significant relationship between using a gas stove in the home and increased prevalence of chronic lower respiratory illness (e.g. asthma) [18-21]. Some data show that self-reported behaviors and housing conditions related to gas stove use are associated with reduced lung function and chronic lower respiratory illness and lower respiratory symptoms in children. For example, previous analyses of NHANESIII found increased prevalence of asthma, chronic bronchitis, and wheeze among children whose parents reported using a gas stove for heat [22,23] or using a gas stove without ventilation [22]. Higher prevalence of childhood ALRI have also been noted in homes with gas stoves compared to homes with electric stoves, however, this finding has not been consistently reported [24-32]. These conflicting results between studies could be related to not taking into account gas stove-related behaviors, such as using it as an auxiliary heat source in the home, or housing conditions known to influence indoor air pollution exposures such as the lack of an exhaust vent near the stove [33-41].

Numerous studies have demonstrated that using a gas stove without ventilation contributes to elevated indoor air pollution concentrations [42-46]. For example, a recent report indicated 62%, 9% and 53% of Southern Californian homes that used gas stoves without ventilation exceeded acute health based standards for NO_2_, CO, and formaldehyde, respectively [47]. In addition, research data has found that using a gas stove as a supplemental heating source, as is done in some homes in the U.S. [48], can lead to elevated concentrations of NO_2_ [46]. Therefore, we hypothesized that children under five years of age who lived in households that used a gas stove without ventilation or where the gas stove was used as a heating source would have an elevated prevalence of pneumonia compared to children who lived in households that used a gas stove with ventilation and only for cooking. We further hypothesized that the prevalence of cough-- a non-specific yet important pneumonia-related symptom-- would also be higher among children who lived in homes that used a gas stove without ventilation or as a heat source.

## Methods

### Study population

The National Center for Health Statistics conducted the Third National Health and Nutrition Examination Survey (NHANES III) from 1988–1994. This is a nationally representative cross-sectional survey of the civilian non-institutionalized U.S. population. Participants were administered standardized interviews in their homes and underwent physical examinations and laboratory testing in mobile examination centers.

The purpose of this analysis was to examine the association between respiratory infections in children under 5 years and behaviors related to the use of a gas stove. Subsequently, this analysis was restricted to children < 5 years of age whose parents: (1) responded to questions about pneumonia and cough, (2) reported that a gas stove was used in the past twelve months in their child’s primary residence, (3) provided information on their use of ventilation when operating the stove, and (4) provided information when asked if they used the gas stove for heat. There were 3,314 children under the age of 5 who lived in homes that reported using a gas stove for cooking. Of those, 3,295 and 3,132 parents provided information on their child’s pneumonia history and cough, respectively. Finally, complete covariate information was available on 3,308 and 3,146 for pneumonia and cough, respectfully.

### Ethics statement

Human subjects approval and documentation of consent for NHANES III child participants was obtained through the National Center for Health Statistics Internal Review Board. Parents or legal guardians of participants under the age of 7 years provided informed consent [49].

### Self-reported respiratory outcomes

Respondents were asked whether their child had pneumonia in the past 12 months (yes/no). This pneumonia variable has been used in two previously published studies that analyzed NHANESIII [50,51]. We also included coughing as a non-specific secondary respiratory outcome that could also be related to respiratory infections. Respondents were also asked if the child had “problems with coughing” in the past 12 months (yes/no) [52].

### Behaviors related to operating gas stoves

Children were assigned into different exposure groups based on how their parent responded to a series of questions related to stove use. Respondents were asked “Is a gas stove or oven used for cooking (yes/no)?” If they reported using a gas stove for cooking, they were then asked: “During the past 12 months was the stove or oven ever used to heat this place (yes/no)?” Respondents that answered *yes* were assigned to the ‘gas stove heating’ group and those who answered *no* were assigned to the ‘no gas heating’ reference group. Additionally, respondents who reported cooking with a gas stove were also asked “Is there an exhaust fan near this stove that sends fumes outside the home (yes/no)?” Respondents that answered *yes* were asked about frequency of exhaust fan usage (never, rarely, sometimes, or always) [52]. Those who reported either not having an exhaust fan or never using the exhaust fan were assigned to the ‘unvented gas stove’ group. Those who answered that they used their exhaust fan rarely, sometimes, or always were assigned to the ‘vented gas stove’ reference group. To evaluate the joint association of venting and heating, we combined these two categories to create mutually exclusive groups that were: (1) heating with an unvented gas stove, (2) heating with a vented gas stove, (3) no heating with an unvented gas stove, and (4) no heating with a vented gas stove (reference group).

### Covariates

Selected characteristics were assessed for their relationship to respiratory outcomes and parental behaviors regarding gas stoves. Covariates were selected by a reviewing the literature [53]. These included: child age and sex, parental race-ethnicity, breast-feeding (never/ever), day care attendance (yes/no), household crowding (<0.5 persons/room, 0.5 - 1 persons/room, and >1 persons/room) [54], maternal smoking during pregnancy (yes/no), environmental tobacco smoking in the home (yes/no), type of heating furnace in the home (none, gas, electrical, oil, propane, other), low birth weight (yes/no), U.S. census region, type of residence (urban versus rural), household income < $20,000, and household referent education level. NHANES interviews and examinations are conducted in the southern states in winter months and northern states in the summer months for operational reasons. We therefore included U.S. census region (Northeast, Midwest, South, West) to not only control for differences in interview time periods, but also to control for known differences in gas stove-related behaviors and pneumonia incidence that are related to geographic regions in the U.S.. We opted to control for socioeconomic status using household income < $20,000 as our income indicator given its low non-response rate (<3%) whereas poverty income ratio had a much higher non-response rate (11%) and respondents differed significantly by race, geographic region, age of the child, and environmental tobacco smoke (all of which are potential confounders themselves) compared to those with these data.

### Statistical analysis

All analyses were conducted using the appropriate sample weights to correct for differential selection probabilities and to adjust for non-coverage and non-response in Stata version 12.1 (StataCorp, College Station, TX). The weighted prevalence (%) and 95% confidence intervals (95% CI) of pneumonia and cough were calculated for the different gas stove usage groups. Chi-square tests assessed the association between health outcomes and the selected covariates, as well as, the relationship between behaviors related to operating gas stoves and select socio-demographic characteristics. Covariates were included in the following multivariate logistic regression models if they were associated with pneumonia or cough at α <0.10 or if previous literature indicated that they were risk factors for pneumonia (e.g. parental educational levels and household income) to control for potential confounding. Subsequently, the models for pneumonia were adjusted for age, race-ethnicity, indoor tobacco smoke, breastfeeding, US Census region, household income under $20,000, and parental education level, whereas the models for cough were adjusted for race-ethnicity, indoor tobacco smoke, breastfeeding, US Census region, household income under $20,000, and parental education level. Separate multiple logistic regression models were used to estimate the odds ratio and 95% CI for each respiratory outcome. Model 1 compared the respiratory outcomes in children from homes that reported using a gas stove for heating to children from homes that did not use a gas stove for heat. Model 2 compared the respiratory outcomes in children from homes where a gas stove was used without ventilation to children from homes where a gas stove was used with ventilation. Model 3 compared the respiratory outcomes in children from homes where: *i*) gas stove was used for heat without ventilation, *ii*) gas stove was used for heat with ventilation, *iii*) gas stove not used for heat without ventilation, and *iv*) gas stove not used for heat and with ventilation (reference group). In addition, we used a chi-squared to test the association between self-reported ventilation and heating with a gas stove.

## Results

For all homes with a gas stove, the weighted prevalence of pneumonia and cough among children under-five years old in NHANES III was 3.23% (95% CI: 2.52, 3.98) and 33.06% (95% CI: 30.81, 35.31), respectively. Among all gas stove users, 12.9% (95% CI: 10.5, 15.3) of the respondents reported using their gas stove for heating purposes and 46.6% (95% CI: 42.2, 51.0) of the participants reported operating their gas stoves without ventilation. Furthermore, participants who did not use ventilation when operating their gas stoves were 2.3 times more likely (p = 0.0004) to report using their gas stove for heating purposes compared to those participants who reported using ventilation when operating their gas stove; thus indicating there was significant overlap between those who used a gas stove for heating and those who did not use ventilation while operating a gas stove.

The crude weighted prevalence of each respiratory outcome by heating and ventilation status in children living in homes with a gas stove is described in Table 1. The crude prevalence of parental-reported pneumonia was higher among children where the respondents did not report using ventilation compared to those who reported using ventilation (4.17% [95% CI: 2.71, 5.63] versus 2.41% [95% CI: 1.64, 3.18], p < 0.05). The crude prevalence of parental-reported pneumonia was also higher among children where the participant reported using their gas stove for heat compared to those who only used their gas stove for cooking (5.65% [95% CI: 2.56, 9.66] versus 2.71% [95% CI: 2.10, 3.49], p < 0.01). Further, the crude prevalence of parental-reported pneumonia was highest among children where the respondents reported using their gas stove without ventilation and for heat compared to those who only used their gas stove for cooking with ventilation (6.83% [95% CI: 3.74, 12.14]) versus 2.31% [95% CI: 1.67, 3.19], p < 0.01). Additionally, child’s age, household referent education level, and U.S. Census region were also significantly associated with parental-reported pneumonia (data not shown). Other covariates, such as income, race-ethnicity, breastfeeding, and environmental tobacco smoke were forced into the model due to their importance in the literature as they relate to childhood ALRIs. Covariates that were significantly associated with cough included environmental tobacco smoke, race-ethnicity, ever breastfed, and region (data not shown). Subsequently, these covariates were included in the following multivariate models. Using a gas heating furnace was not included in the final models since this variable was not related to either respiratory outcome in our study and there was no evidence for confounding attributable to homes using a gas furnace.

**Table 1:** **Prevalence of ALRI among children <5 years stratified by gas stove ventilation and heating status**

	**Pneumonia**			**Coughing**		
	**N**	**%**	**95% CI**	**N**	**%**	**95% CI**
All	120	3.24	2.52, 3.98	1,144	33.06	30.81, 35.31
**Was ventilation used when operating gas stove**						
Yes	48	2.41	1.64, 3.18	567	32.51	28.33, 36.69
No	72	4.17*	2.71, 5.63	574	33.40	28.99, 37.80
**Was gas stove used for heating**						
Yes	86	5.65**	2.56, 9.66	231	40.34	31.86, 49.43
No	32	2.71	2.10, 3.49	908	31.80	29.16, 34.57
**Heating and ventilation status combined**						
Unvented and Heat	25	6.83**	3.74, 12.14	157	44.02	34.22, 54.31
Vented and Heat	7	3.54^a^	1.56, 7.80	74	33.73	22.02, 47.84
Unvented and only cooking	45	3.23	2.12, 4.89	412	30.87	27.01, 35.03
Vented and only cooking	41	2.31	1.67, 3.19	493	32.46	28.32, 36.88

Abbreviations: CI, confidence interval.

*P < 0.05.

**P < 0.01.

^a^May be statistically unreliable, relative standard error (SE/percent) >30%.

The association between using a gas stove for heat and each respiratory outcome adjusted for confounders is described in Table 2. After adjusting for other risk factors, the adjusted odds of pneumonia in the past 12 months was twice as high (aOR: 2.08, 95% CI: 1.08, 4.03) among children under 5 years of age who lived in a household where gas stoves were used for heat compared to those who resided in homes where gas stoves were only used for cooking. Similarly, the odds that children had a problem with coughing in the past 12 months was significantly higher (aOR: 1.66, 95% CI: 1.14, 2.43) among children who lived in a household where the participant reported using their gas stove for heat compared to children residing in homes where gas stoves were only used for cooking after adjusting for other risk factors.

**Table 2:** **Odds ratios for ALRI among children <5 years old: gas stove heating versus cooking only**

	**Crude OR**	**95% CI**	**Adjusted OR**	**95% CI**
Pneumonia^c^	2.15	1.25, 3.70	2.08^a^	1.08, 4.03
Cough^c^	1.45	0.99, 2.12	1.66^b^	1.14, 2.43

^a^Model adjusted for age, parental education, census region, race, household income < $20,000, ever breastfed, and environmental tobacco smoke (N = 3,240).

^b^Model adjusted for parental education, census region, race, household income < $20,000, ever breastfed, and environmental tobacco smoke (N = 3,084).

^c^The reference group for each comparison are homes that reported cooking only heating.

The association between using a gas stove without ventilation on each respiratory outcome after adjusting for confounders is presented in Table 3. After adjusting for other risk factors, the odds that children under 5 years of age had parental-reported pneumonia in the past 12 months was significantly greater (aOR: 1.76, 95% CI: 1.04, 2.98) among children who lived in a household where the participant reported that they did not use ventilation when operating their gas stove compared to children residing in homes that used ventilation when operating gas stoves. The association between parental-reported coughing was not significantly different between those who reported not using ventilation and those who did report using ventilation after adjusting for other risk factors (aOR: 1.23, 95% CI: 0.87, 1.75).

**Table 3:** **Odds ratios for ALRI among children <5 years old: no gas stove ventilation versus ventilation**

	**Crude OR**	**95% CI**	**Adjusted OR**	**95% CI**
Pneumonia^c^	1.76	1.09, 2.83	1.76^a^	1.04, 2.98
Cough^c^	1.04	0.77, 1.40	1.23^b^	0.87, 1.75

^a^Model adjusted for age, parental education, census region, race, household income < $20,000, environmental tobacco smoke, and ever breastfed (N = 3,254).

^b^Model adjusted for parental education, census region, race, household income < $20,000, ever breastfed, and environmental tobacco smoke (N = 3,099).

^c^The reference group for each comparison are homes that reported using ventilation.

**Figure 1:**
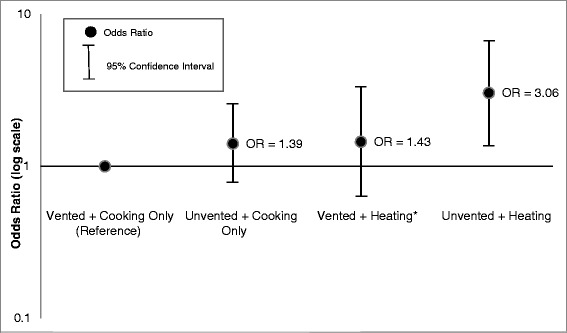
**Odds ratios for Pneumonia among children <5 years old: comparison of heating and ventilation combined.** *May be statistically unreliable, relative standard error (SE/percent) >30%.

**Figure 2:**
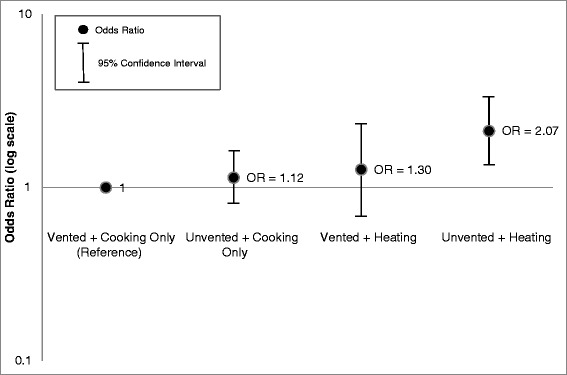
**Odds ratios for Cough among children <5 years old: comparison of heating and ventilation combined.**

The joint association of ventilation and heating are described in Figures 1 and 2. Compared to children residing in homes where the participants reported only using gas stoves for cooking and with ventilation, the odds of parental-reported pneumonia were three times higher (aOR: 3.06, 95% CI: 1.32, 7.09) among children who lived in homes where the gas stoves were used for heat without ventilation after adjusting for confounders (Figure 1). The odds of a problem with parental-reported coughing was also significantly higher among this group of children who lived in homes where gas stoves were used for heat without ventilation (aOR: 2.07, 95% CI: 1.29, 3.30) after adjusting for confounders (Figure 2). Compared to the reference group, the adjusted odds of parental-reported pneumonia and cough were not significantly different for children residing in a home where the participant reported using their gas stove for heat with ventilation or not using ventilation when using their gas stove for cooking only (Figures 1 and 2).

## Discussion

This study observed that the prevalence of pneumonia and problems with coughing in U.S. children less than 5 years of age were significantly associated behaviors related to operating gas stoves; namely using it for heat without ventilation. While each of these behaviors was associated with an increased likelihood of respiratory illness in this selected population, the odds of pneumonia and a problem with coughing was significantly higher only in children who resided in homes where gas stoves were used for heating without ventilation. This observation is important because ventilation and using a gas stove for heat are modifiable factors and provide an opportunity for public health intervention. For instance, approximately 90% of the respondents in this sample who did not use ventilation reported the absence of an exhaust vent near their stove. This suggests that significant infrastructure improvements regarding ventilation, beyond just behavior modification, are needed to improve health outcomes related to ventilation and gas stoves [55]. Moreover, and not surprisingly, we found that using a gas stove for heating or without ventilation occurred at greater frequency in lower socioeconomic status households as well as among African American households, even after adjusting for age of the home and census region (data not shown). The degree to which gas stove-related behaviors and housing conditions are a source of respiratory health disparities in the United States is unclear yet our findings suggest that it deserves further attention.

While this study is the first to examine the nation-wide association between behaviors related to operating gas stoves and pneumonia prevalence in U.S. children, there is considerable evidence from epidemiological studies in developed countries that gas stoves used for cooking, heat or without ventilation are associated with an increased risk of chronic lower respiratory illnesses and symptoms in children [56-63]. Behaviors related to gas stove use has also been shown to be related to acute lower respiratory illness in young children. A cross-sectional study (n = 426) conducted in Hong Kong reported a dose–response relationship between the frequency of gas cooking, an indicator of gas stove-use intensity, and the prevalence of respiratory illnesses among children ≤ 6 years who lived in a sector of the city with the lowest ambient air pollution [40]. Specifically, in homes where a gas stove was used for cooking ≥3 meals per day the likelihood of pneumonia or bronchitis was 2.6 times higher (OR = 2.6; 95% CI: 1.3, 5.4) compared to children from homes where gas stoves were used for ≤1 meal per day [40]. This study also found that children from homes with unvented gas stove were 3.7 times more likely to have pneumonia or bronchitis (OR = 3.7; 95% CI: 0.8, 16.7) compared to children from vented gas stove homes. MacIntyre et al. [64] examined the dose–response relationship between air pollutants which are also known to be emitted from gas stoves reported that the odds of pneumonia ORs increased by 30% and 76% with every ten ppb increase in NO_2_ and PM_10_. Another study that compared unvented gas heaters to vented gas heaters found an Odds Ratio of 1.16 (95% CI: 1.01 - 1.34) for cough among school-aged children [29]. A recent home intervention study by Paulin et al. [65] found that adding an exhaust vent to a gas stove reduced indoor NO_2_ concentrations by 9.6 ppb on average. Interestingly, the magnitude of the effects observed in our study is comparable to the effect estimates described above [29,40,64].

In light of recent research data that point to the importance of ventilation to reduce indoor air pollution such as nitrogen dioxide when operating a gas stove [47,65-67], it is important to consider the biologic plausibility that gas stove emissions could be related to ALRI. Firstly, data from epidemiologic research suggest that increasing nitrogen dioxide exposure is likely to increase risk of lower respiratory symptoms and ALRIs [29,31,44,64,68-75]. Experimental toxicological studies show that exposing bronchial epithelial cells to nitrogen dioxide causes oxidative stress [76], production of pro-inflammatory molecules [77,78], suppression of the innate response [79,80], impaired macrophage activation [81], and altered function of surfactant proteins which are critical in phagocytosis and inflammation processes [76]. Such effects may result in reduced host defenses against spread of viral or bacterial pathogens that cause ALRI [79-82]. An epidemiological study found that children of mothers who cook with a gas stove were significantly more likely (OR = 17.1, 95% CI: 3.0-98.1) to spontaneously release TNF-α [83], an important indicator of an immune response to acute viral infections [84]. A separate study of children under 24 months found that the presence of a gas stove in the home was associated with a 46.5% increase (p < 0.01) in T-helper 2 cytokines [85]. Moreover, an imbalance of T-helper 2/T-helper 1 cytokines has been associated with more severe cases of lower respiratory viral infections in young children [86] and in vivo experiments have shown that increased T-helper 2 cells were associated with increased susceptibility to pneumococcal pneumonia in a mouse model [87]. Taken together, these studies support a biological plausibility for our findings.

While our study has many strengths including the ability to adjust for multiple confounders in a large, nationally-representative sample of U.S. children, there are several limitations to this study that warrant consideration. Namely, these data are cross-sectional which prevents us from interpreting the temporality of the observed associations. Also, we relied upon parental-reported health outcomes in the past 12 months. Given this long recall period, recall bias is a potential limitation and may impact our findings. For example, if parents who reported not using ventilation or whose homes did not have an exhaust vent, were more likely to recall one of these respiratory illnesses in their child than parents who reported using ventilation our estimates may be biased away from the null and overestimate the association. Alternatively, if parents who did not report using ventilation or whose homes did not have an exhaust vent were less likely to recall one of these respiratory illness in their child, our estimate may be biased towards the null which would lead to an underestimate of the observed effects. If the recall bias between parents using or not using a gas stove for heating (or ventilation), there may be non-differential bias and our estimate may also be attenuated. It is also possible that parents who recalled that their children had pneumonia were also more likely to recall that their child had problems with coughing.

Another limitation is how we chose to group ventilation. Respondents were asked how often they used their ventilation (i.e. never, rarely, sometimes, always) when operating their gas stove. We opted to combine participants who rarely, sometimes and always used their exhaust vent into one category and compare them to those who never used their exhaust vent or did not have an exhaust vent near their gas stove. This binary approach likely includes some degree of misclassification given the ambiguity between the less frequent terms of use. However only 6 people reported never using their vent and 6 people reported rarely using their vent so the error contributed from this misclassification is likely to be small. It is also worth noting we adjusted for socioeconomic status using a categorical income variable that dichotomized participants as having a household income > $20,000 and respondent education level. These covariates were chosen because they had the highest response rate and addressed two important factors related to poverty. Another limitation of our study was that we were unable to compare the effects of stove operating behaviors on respiratory illnesses in homes that used electric stoves since only respondents with a gas stove were asked about ventilation practices and using their stove for heat. Therefore, our study may only be generalizable to children residing in homes with a gas stove. In addition, there were no indoor air pollutants measured in NHANES which could be used to further characterize exposure. Also, these data are more than 25 year old and thus may not accurately reflect the current status of gas stove use behaviors. However, gas stoves remain commonplace in the U.S. [88]and more recent research suggests heating with a gas stove and poor ventilation is still prevalent in the US [46,47,89]. For instance, one study conducted in California found that among those who reported using a cooking appliance, as much as 61% did not use ventilation while cooking [89]. Also, we cannot discount the influence of unmeasured confounders that are likely to exist (i.e. climate).

## Conclusion

In conclusion, we observed that U.S. children less than five years old who live in households with a gas stove which is used for heat without ventilation have the highest prevalence of pneumonia and cough. Additionally, using a gas stove for heat or without ventilation was also associated with higher prevalence of pneumonia and cough. These behaviors or housing conditions may be amenable to public health intervention due to their modifiable nature. More research is needed to establish the causality of these observations particularly as a driver of environmentally-determined health disparities. Also, since NHANESIII is now more than twenty years old and currently represents the only U.S. population-based data set to examine the relationship between gas stove usage and respiratory illnesses, we recommend that future NHANES cycles incorporate such questions again so that health science researchers can evaluate whether circumstances have changed in the United States.

## Abbreviations

ALRI: Acute lower respiratory infection
CI: Confidence interval
NHANES III: Third National Health and Nutrition Examination Survey
OR: Odds ratio
